# The Perception of Stress Pattern in Young Cochlear Implanted Children: An EEG Study

**DOI:** 10.3389/fnins.2016.00068

**Published:** 2016-03-08

**Authors:** Niki K. Vavatzanidis, Dirk Mürbe, Angela D. Friederici, Anja Hahne

**Affiliations:** ^1^Max Planck Institute for Human Cognitive and Brain SciencesLeipzig, Germany; ^2^Saxonian Cochlear Implant Center, Department of Otorhinolaryngology, Technische Universität DresdenDresden, Germany

**Keywords:** cochlear implants, children, deafness, stress pattern, mismatch response, EEG/ERP, auditory perception, language acquisition

## Abstract

Children with sensorineural hearing loss may (re)gain hearing with a cochlear implant—a device that transforms sounds into electric pulses and bypasses the dysfunctioning inner ear by stimulating the auditory nerve directly with an electrode array. Many implanted children master the acquisition of spoken language successfully, yet we still have little knowledge of the actual input they receive with the implant and specifically which language sensitive cues they hear. This would be important however, both for understanding the flexibility of the auditory system when presented with stimuli after a (life-) long phase of deprivation and for planning therapeutic intervention. In rhythmic languages the general stress pattern conveys important information about word boundaries. Infant language acquisition relies on such cues and can be severely hampered when this information is missing, as seen for dyslexic children and children with specific language impairment. Here we ask whether children with a cochlear implant perceive differences in stress patterns during their language acquisition phase and if they do, whether it is present directly following implant stimulation or if and how much time is needed for the auditory system to adapt to the new sensory modality. We performed a longitudinal ERP study, testing in bimonthly intervals the stress pattern perception of 17 young hearing impaired children (age range: 9–50 months; mean: 22 months) during their first 6 months of implant use. An additional session before the implantation served as control baseline. During a session they passively listened to an oddball paradigm featuring the disyllable “baba,” which was stressed either on the first or second syllable (trochaic vs. iambic stress pattern). A group of age-matched normal hearing children participated as controls. Our results show, that within the first 6 months of implant use the implanted children develop a negative mismatch response for iambic but not for trochaic deviants, thus showing the same result as the normal hearing controls. Even congenitally deaf children show the same developing pattern. We therefore conclude (a) that young implanted children have early access to stress pattern information and (b) that they develop ERP responses similar to those of normal hearing children.

## Introduction

Language acquisition is a marvelous thing: An infant arrives quite naïve into our world of sound and language and successfully accomplishes the task of attaching meaning to the streams of auditory information that is speech. In this process of turning sound into meaningful utterances, one of the most important steps the infant has to master is finding the word boundaries so as to have units to which meaning can be attached (Newman et al., 2006). This is not a trivial task, since words in spoken language are not necessarily flanked by pauses, unlike written words that are flanked by whitespaces. That the knowledge of boundaries is specific to a particular language becomes obvious when listening to a lengthy utterance in a foreign language, preferably one has never heard before: The listener will have a hard time identifying the words. This raises the question, how infants learn where the word boundaries in their native language are.

Fortunately, infants are brilliant in grasping regularities (Mueller et al., 2012; Krogh et al., 2013) and language offers rule-based cues for the identification of word boundaries. Phonotactic rules restrict the place where a word boundary may be (Friederici and Wessels, 1993; Mersad and Nazzi, 2011; Rossi et al., 2011; Graf Estes, 2014), as certain phoneme combinations are simply not valid at the beginning or at the end of word for a specific language. For example, German phonotactic rules do not allow the segmentation of “lautes Kreischen” (German for “loud screech”) into [laute |skreischen], because/skr/ is not a valid word onset phoneme combination in German, whereas it is perfectly acceptable in English (e.g., “screech” or “screen”).

Other cues for word boundaries may come from prosody. Stress pattern, for example, can mark the beginning or end of a word in rhythmic languages like German or English. Both German and English are characterized by a predominant trochaic meter (Cutler and Carter, 1987), that is, the majority of disyllabic words is stressed on the first syllable. Thus stress can convey the strong suggestion to segment the German phrase “letztensommergingenmeineelternwandern” into “ ′letzten ′Sommer ′gingen ′meine ′Eltern ′wandern” (“last summer my parents went hiking”), which is the correct form.

The perception of the overall stress pattern of the native language seems to be present from very early on, as newborns were shown to follow the native stress pattern already in their cries: Whereas German infants tend to stress the beginning of their cries, French infants stress the latter part (Mampe et al., 2009; French not being a rhythmic language but having more occurrences of words with iambic stress, i.e., disyllables that are stressed on the last syllable). Behavioral experiments also demonstrated a listening preference for the native stress pattern in 6-month-old German infants (Höhle et al., 2009) and 9-month-old English infants (Jusczyk et al., 1993).

When learning a language with a strong metric bias, relying on stress pattern for word segmentation is apparently a useful starting rule for identifying words in fluent speech. A series of behavioral experiments by Jusczyk et al. (1999) could demonstrate the importance of stress pattern for word segmentation in the early phases of language acquisition. 7.5-month-old English infants could detect trochaic words in fluent speech but failed to do so with iambic words. As infants grow older, they refine their set of rules and thus rely less on stress pattern. The same series of experiments by Jusczyk et al. (1999) showed that by the age of 10.5 months the infants are able to correctly segment iambic words as well.

Electrophysiological studies confirm the early sensitivity of infants toward stress pattern. At 4–5 months of age the electrophysiological response of German infants already differentiate between iambic and trochaic stress patterns in a language specific manner (Weber et al., 2004; Friedrich et al., 2009). The pattern of the event-related potential (ERP) reflects the habituation to the native stress pattern with German infants showing a positive mismatch response (MMR) toward the foreign iambic stimulus, and French infants display it toward the non-native trochaic stress pattern (Friederici et al., 2007).

The importance of such cues for language acquisition becomes evident in children that are less sensitive to them. Several studies suggest that children with language impairments, like language impairment (SLI) or dyslexia, are not as sensitive to auditory features like duration, rise time, frequency, etc., as children with normal language development (Benasich et al., 2002; Corriveau et al., 2007; Leppänen et al., 2010). For stress pattern specifically, Goswami et al. (2013) showed that 9- to 13-year-old dyslexic children perform poorly in syllable stress tasks compared to controls. An electrophysiological study by Friedrich et al. (2004) showed that already 2-month-old infants at risk of SLI show a diminished response toward differences in vowel duration (longer duration being a mark of a stressed syllable) when compared to controls. Likewise, children who score low in word and sentence production at the age of 2.5 years differ from typically developing children in their electrophysiological responses at the age of 4 to 5 months when listening to stress pattern differences (Friedrich et al., 2009). In contrast, children who display good segmentation skills in their first year of life have a better expressive vocabulary at the age of two and have higher language scores even as pre-schoolers (Newman et al., 2006).

The cochlear implant (CI) has given children with severe to profound sensory hearing loss access to the auditory world with great success by directly stimulating the auditory nerve and thus bypassing the dysfunctioning inner ear. Yet, stimulating the auditory nerve with up to 22 active electrodes cannot be compared to the stimulation by thousands of inner ear hair cells. Consequently, frequency discrimination with the CI is lower (Zeng et al., 2014) and the dynamic range smaller (Zeng, 2004), even though technical innovations strive to close the gap. If missing the auditory cues for word segmentation hinders normal language acquisition as described for children with SLI or dyslexia, what does that mean for infants who receive the diminished input of a CI? Does the implant transmit sufficiently detailed auditory cues and which ones?

A large number of studies describes language outcomes of children with a cochlear implant after several years of implant use (e.g., Svirsky et al., 2000; Geers et al., 2003; Niparko et al., 2010; Dunn et al., 2014; Kronenberger et al., 2014; Faes et al., 2015; van Wieringen and Wouters, 2015) and report variable but also encouraging results (e.g., Geers and Sedey, 2011) show that around 70% of implanted adolescents have age appropriate language skills). Yet, little is known about the perceptual resources these children have during the phase of acquisition. This, however, would be crucial information as the window of language acquisition is finite (Lenneberg, 1967) and implanted children are already delayed in receiving appropriate auditory input. Thus, the earlier and more we know about what an implanted child can extract from its language environment, the better and more specific therapeutic intervention can become and the better we could acknowledge possible compensatory strategies and the potential additional cognitive resources that are needed.

A stressed syllable is characterized by longer duration, higher amplitude (loudness) and a change of the fundamental frequency (pitch; Fry, 1958). As to the longer duration, we were able to show that congenitally deaf children need only 2 months of hearing experience with the implant to differentiate between short and long vowel durations. After 4 months, they have already reached the level of normal hearing peers (Vavatzanidis et al., 2015). We can thus conclude that at least one prerequisite for the perception of stress pattern is available to the children soon after implantation. As to amplitude and fundamental frequency, we mentioned above that cochlear implants have limitations in the dynamic range (affecting amplitude) and the spectral range (affecting the fundamental frequency). Postlingually deafened adult CI users show a diminished sensitivity for the fundamental frequency (Rahne et al., 2011) but are still able to use stress pattern for lexical segmentation with the help of the other cues (Spitzer et al., 2009). The question remains open, however, whether prelingually deafened children master stress pattern discrimination with the diminished input of the CI.

The ability of children's stress perception has been the focus of speech perception studies since the early days of the cochlear implant. Thielemeir et al. (1985) report that with the single-electrode implant 80% of the tested children could differentiate between a monosyllable and a spondee (a disyllabic word with equal stress on both syllables) and 60% could even perceive the difference between a trochee (a disyllable with the stress on the first syllable) and a spondee, whereas prior to implantation only 40% of the children could perceive the voice of the experimenter despite a hearing aid. Stress pattern perception (among other features) further improved with the introduction of multi-channel cochlear implants as demonstrated by Osberger et al. (1991) who tested both multi-channel and single-channel implant users. A number of factors, however, complicate the interpretation of former studies with regard to the children's abilities to extract the native stress pattern.

First of all, the studies vary greatly with respect to the age of their participants (infants—adolescents), their age at implantation (infancy—late adolescence), their amount of experience with the CI (months to several years) and the onset of deafness (congenital—prelingual—perilingual—postlingual). Also, most studies stem from the nineties (Thielemeir et al., 1985; Osberger et al., 1991; Miyamoto et al., 1996; Fryauf-Bertschy et al., 1997; Tyler et al., 1997) and since then, implants and speech processors have improved vastly, providing better speech perception. Furthermore, most of these studies employed the Monosyllable, Trochee, Spondee (MTS) test (Erber and Alencewicz, 1976) to determine stress pattern recognition abilities. The MTS consists of 12 pictured nouns: four monosyllables, four trochees and four spondees. Upon hearing a noun, the child points to one of the pictures. Each noun is presented twice in a session. If the child chooses a wrong pictured word that has the correct stress pattern, it still scores a point in stress perception. While the MTS is a commonly used tool for the evaluation of speech perception and employed also nowadays with adult CI users (e.g., De Ruiter et al., 2015), one problem of the test is that it confounds stress pattern perception with the number of syllables in the case of the monosyllables. Another problem is that it can be only performed with children old enough to understand the task and point at the correct picture and who have already acquired a certain set of vocabulary. Finally, in order to determine whether children with a cochlear implant actually perceive the dominance of a certain stress pattern in their native language such that they may employ it as a tool for word segmentation, a more vigorous experimental setup contrasting the native with another stress pattern would be desirable.

Tasks more suitable for younger children are the visual habituation procedure and the preferential looking paradigm used in many stress pattern perception studies of normal hearing children (e.g., in the seminal studies by Peter Jusczyk) and employed by the few recent studies on stress pattern with implanted children. In the visual habituation procedure, the child hears one item repeatedly while looking at a screen. When the looking time toward the screen decreases, a novel item is presented and the change in looking time is evaluated. This has the benefit of being not only suitable for infants who are still in the early phases of language acquisition, but also suitable for testing a wide range of acoustic changes.

Core et al. (2014) tested six prelingually deafened children aged 3;4-5;5 years with 1-4 years of implant experience with the stress pattern contrast /'beibi/ vs. /bə, bi/. A Bayesian linear regression model could detect significant increases in looking time for novel trials in four of the six children. However, as the stimulus pair differed not only in the position of stress but also in the vowel quality of the first syllable, it is not clear whether the effect can be attributed entirely to the discrimination between iambic and trochaic stimuli. Segal et al. (2016) also tested prelingually deafened children with the visual habituation procedure. Twenty participants who received their implant between 10 and 28 months were tested during the first 6 months of implant use. All children grew up in monolingual Hebrew-speaking environments, where the predominant stress pattern is iambic. The stimulus was the pseudoword/doti/ stressed either in a trochaic or in an iambic pattern (/dóti/ vs. /dóti/). The results show that the implanted children had longer looking times for novel trials and that the effect was most pronounced when the stimulus changed from the foreign stress pattern to the native iambic stress pattern.

Taken together, there is only scarce information on a) whether implanted children perceive and acquire a sensitivity toward their native stress pattern that may aid them to segment fluent speech into single words and b) whether the perception is present from the first day on or whether it evolves over time. On that ground we performed the present study that compares the ERPs of implanted children and normal hearing peers elicited by the native trochaic rhythm vs. the non-native iambic rhythm. We specifically used ERPs, as they offer several advantages. By measuring electrophysiological data, we obtain a direct and objective measure of ongoing brain processes without depending on any overt response of the child (e.g., pointing at the correct picture). This is important, as we observe that some congenitally deaf children lack auditory attention in the first weeks of implant use. That is, they rarely react to salient or loud stimuli, though it is clear from some isolated reactions that they are able to perceive them. Whereas in behavioral studies this would pose a problem, the ERP components will show whether the stimuli have been processed despite the lack of an overt behavioral reaction. Also, both behavioral and EEG/ERP studies suffer when the child is overactive or fussy and will not engage in a particular task. In our study, the oddball paradigm was to our great advantage as it works even when the participant does not pay any attention to the stimuli *per se*. Instead, we could engage the child in silent play by watching a picture book or a movie, playing with puppets, etc.

## Methods

### Participants

One of the greatest challenges in clinical research is to arrive at a homogenous group of participants. Studies of cochlear implants have to address a wide range of variables that have potential influence on the research outcome. For children, the effect of *age at implantation* on later language development is perhaps the most widely discussed. Adverse maturational effects (e.g., unfavorable reorganization of the auditory cortex) are less probable if implantation occurs early and children are still in their natural phase of language acquisition. In addition, the earlier the implantation, the smaller the gap that a child has to close to reach its age peers. While there is no definite conclusion on the exact age range optimal for implantation, most studies agree that implantation of prelingually deafened children implanted after the age of about four severely reduces the chance on normal cortical maturation and successful language acquisition with even worse outcomes if implantation occurs after the age of about seven years (e.g., Tyler et al., 1997; Sharma et al., 2007; Szagun, 2010). We therefore limited our subject group to children implanted up to the age of four (max. 50 months). Hearing performance changes with growing auditory experience due to the implant. We therefore chose a longitudinal design to monitor the progress in stress pattern perception over the first 6 months in bimonthly intervals. Thus, the factor *time of implant use* was a direct variable of our analysis. Also, as any *previous experience of auditory stimulation* works in favor for subsequent language acquisition, we performed a separate analysis for the congenitally deaf children. Gordon et al. (2010) have furthermore shown that in the case of sequential implantation a short *interimplant delay* leads to normal cortical activity (e.g., P1), whereas long interimplant delays (more than 2 years) show abnormal lateralizations in stimulus processing. The majority of our participants were implanted simultaneously. The four sequentially implanted children in our study had interimplant delays between two and 7 months, so that adverse effects due to asymmetric maturation should be minimal or absent. The *speech processing strategies* will vary between companies and will influence to a certain degree the speech perception and thus the performance in stress pattern recognition. This, however, is a variable that cannot be controlled as even for the same implant and speech processor, several processing strategies are available and are chosen with regard to the current environmental (e.g., noisy street vs. quiet home hours). Additional settings (e.g., microphone sensitivity, balance between high, and low frequencies, etc.) may change each time the child is seen at our center, such that processor strategies and settings are a variable of potential influence but in practice beyond control in such a study.

#### Children with cochlear implants

Nineteen bilaterally hearing impaired children, who received a cochlear implant participated in the study. For all children, CI indication was confirmed by pediatric audiological assessment consisting of a brain stem electric response audiometry (BERA) and subjective audiometry. When a period of bilateral hearing aid use proved to be without benefit, cochlear implantation was performed on both ears (see details in Table 1). Two children had to be excluded from further analysis due to excessive artifacts. Of the remaining 17 children, eight were congenitally deaf. The other nine children had severe or profound hearing loss with some residual hearing prior to implantation, which was deemed insufficient for language acquisition in repeated assessments by audiologists and speech therapists despite the use of hearing aids.

**Table 1:** **Details for the implanted children entering the final analysis**.

**Child**	**Sex**	**Mode**	**Implant**	**Processor**	**Age@activation**
1	m	Simultaneous bilateral	CI512	CP810	32
2	m	Simultaneous bilateral	Concerto	Opus2	24
^*^3	m	Simultaneous bilateral	Concerto	Opus2	15
4	m	Sequential bilateral	Concerto	Opus2	25/27
5	m	Sequential bilateral	CI422 & CI512	CP810	50/57
^*^6	m	Sequential bilateral	CI422	CP810	11/15
^*^7	m	Simultaneous bilateral	CI512	CP810	37
^*^8	m	Simultaneous bilateral	CI422	CP810	11
^*^9	f	Simultaneous bilateral	Concerto	Opus2	21
10	f	Simultaneous bilateral	HiRes90K Advantage	Harmony	31
^*^11	m	Sequential bilateral	Concerto	Opus2	12/15
12	f	Simultaneous bilateral	CI422	CP810	39
13	f	Simultaneous bilateral	Concerto	Opus2	11
^*^14	f	Simultaneous bilateral	Concerto	Opus2	14
15	f	Simultaneous bilateral	Concerto	Opus2	9
16	m	Simultaneous bilateral	HiRes90K	Naida	11
^*^17	m	Simultaneous bilateral	CI522	CP910	11

*Age at activation given in months (second age reference refers to the second implant of the sequentially implanted children). Asterisks mark the congenitally deaf children*.

After implantation, all children entered the rehabilitation program at the Saxonian Cochlear Implant Center, University Hospital Dresden, Germany. There they received a bimonthly fitting of the speech processor and multidisciplinary speech and language therapy for up to 3 years. The first activation occurred 1 month postsurgically during a 5-day rehabilitation stay. Age at first activation of the implant ranged from 9 to 50 months (*M* = 22 months, *SD* = 13 months, *Mdn* = 18 months).

The EEG recordings were performed longitudinally at the regular bimonthly rehabilitation stays: in the week of initial activation (M0), after two (M2), four (M4), and six (M6) months of implant use plus an additional pre-operative measurement serving as baseline (Mpre). Not all of the above recordings could be obtained for each child due to occasional illness or restlessness of the child. Size and age distributions of the groups are listed in Tables 2, 3.

**Table 2:** **Number of participants and age (in months) of final groups**.

	**Mpre**	**M0**	**M2**	**M4**	**M6**
N	8	9	8	11	11
Range	8–29	11–39	13–27	12–44	14–56
Median	10	14	14	26	30

**Table 3:** **Number of participants and age (in months) of the subgroups of congenitally deaf children**.

	**Mpre**	**M0**	**M2**	**M4**	**M6**
N	5	5	5	5	4
Range	8–19	11–21	13–23	16–40	18–42
Median	10	12	14	16	24

#### Normal hearing controls

Two control groups of normal hearing (NH) full-term children were measured at the Max Planck Institute for Cognitive and Brain Sciences in Leipzig, Germany. The first group NH1 (*N* = 12) matched the CI children of group Mpre in age and gender at the time point of measurement (5 female, age range = 9–29 months, *Mdn* = 10 months, *SD* = 6.85). Likewise, the second group NH2 (*N* = 12) matched age and gender of the implanted children included in M4 (4 female, age range = 12–44 months, *Mdn* = 26 months, *SD* = 10.72).

No additional control group matched for hearing age was obtained as the original study of this paradigm (Weber et al., 2004) already provides data of 4- to 5-months old infants tested with the same stimuli.

For all children (implanted and controls), informed consent was signed by a parent or a person having the custody for the child. The following procedures were approved by the local ethics committee (Medical Faculty Carl Gustav Carus of the Technische Universität Dresden).

### Stimuli and procedure

**Figure 1:**
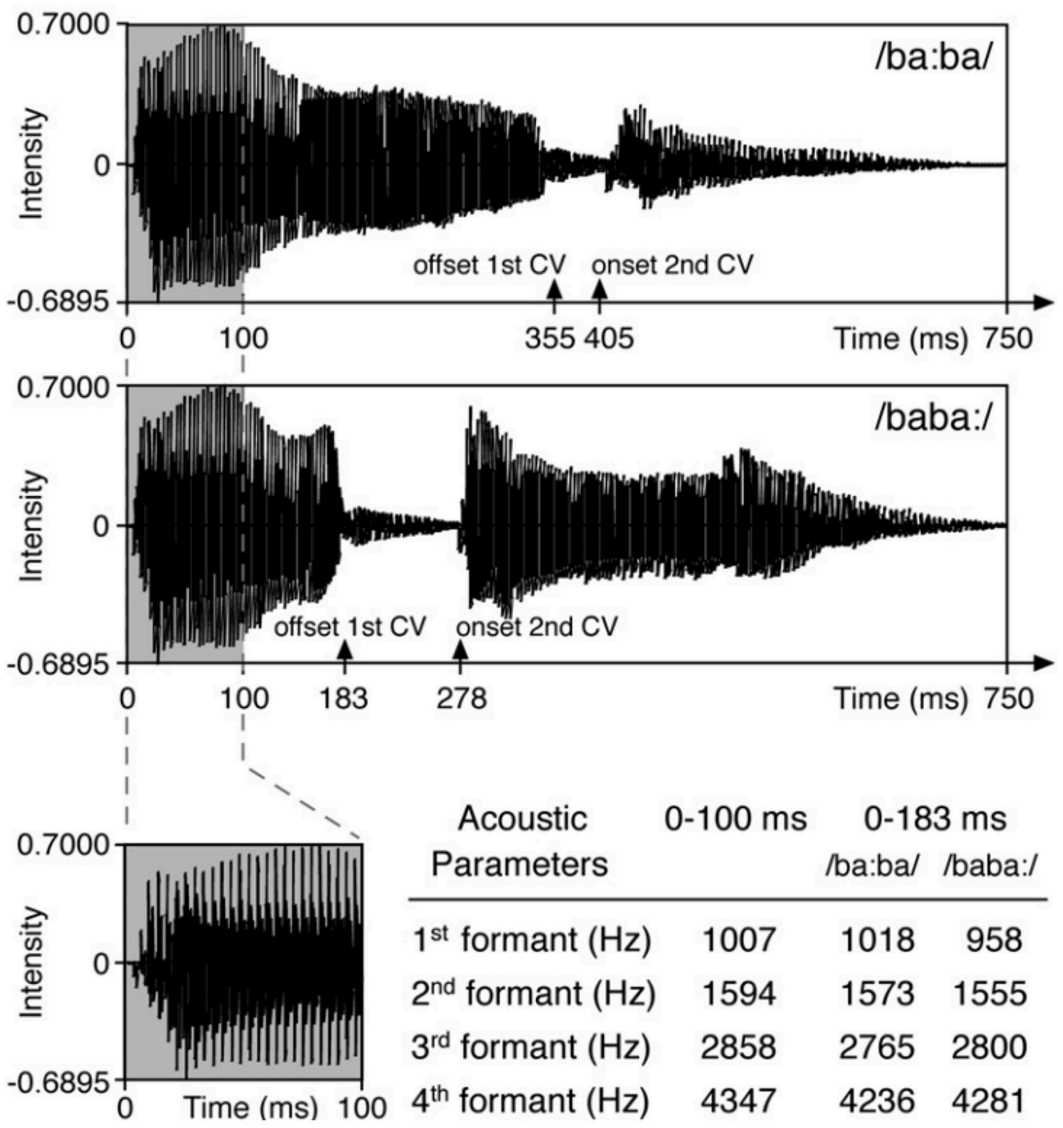
**Stimuli of the original study by Weber et al. (2004)**. Reprinted from Friederici et al. (2007) with permission from Elsevier.

Stimuli and paradigm originate from the study by Weber et al. (2004) (Figure 1). In a random oddball paradigm the disyllabic pseudoword/baba/ was presented at 65 dB SPL in infant directed speech stressed either on the first syllable (/ba:ba/ = trochaic rhythm, native stress pattern in German) or on the second syllable (/baba:/ = iambic rhythm). The stimuli were kept identical in the first 100 ms, such that any acoustic difference between the stimuli was as close as possible to the onset of stress difference. This was realized by replacing the first 100 ms of the trochaic stimulus by the first 100 ms of the iambic stimulus. German native speakers judged both stimuli as sounding natural. In one of two blocks the trochaic syllable was the standard stimulus (frequency of 5/6) and the iambic syllable the deviant stimulus (1/6) and vice versa. The order of the two blocks was pseudorandomized across an individual's session and across all participants. Deviants were separated by two to seven standard stimuli. Each block contained 600 trials with an interstimulus interval (ISI) of 855 ms. Stimuli were presented with the software Presentation® (NeuroBehavioral Systems, Albany, CA). During the auditory stimulation via loudspeakers, children were sitting awake on their parent's lap watching a silent animated movie or being silently entertained with books, puppets, etc., by one of the experimenters.

### EEG recording

Data were obtained continuously with Ag-AgCl^−^ electrodes positioned according to the International 10-20 System in an elastic electrode cap (EasyCap, GmbH, Herrsching, Germany). Nine scalp sites (F3, Fz, F4/C3, Cz, C4/P3, Pz, P4) and the left and right mastoid were recorded. In some cases where the position of the speech processor hindered the correct placement of the mastoid electrodes the speech processor was removed from the ear and taped onto the cap as close as possible to the original position but ensuring that proper placement of the mastoid electrodes was possible.

An electrooculogram was obtained from two horizontal electrodes at the outer canthi of the left and right eye and from a vertical electrode above the right eye. An additional vertical electrode was recorded below the right eye whenever possible. It was omitted if otherwise the child would not have tolerated the EEG measurement. The signal was sampled at 500 Hz and amplified with a PORTI-32/MREFA (Twente Medical Systems, Oldenzaal, The Netherlands) with electrode Cz as online reference.

Data were downsampled offline to 256 Hz and rereferenced to the average of both mastoids. If one mastoid was too corrupted by artifacts, the other mastoid served as single reference. A band-pass filter of 1–15 Hz reduced slow drifts and muscle artifacts. Trials with the signal at the midline electrodes (Fz, Cz, Pz) or eye electrodes exceeding 80 μV within a 200 ms sliding window were rejected. A subsequent correction of eye blinks and eye movements was applied (EEP 3.2.1, developed by the CBS MPI, Leipzig, Germany and distributed by ANT Neuro, Enschede, Netherlands). The standard trial immediately following a deviant trial was removed from analysis. All sessions had a minimum of 50 deviants (50%) and 200 standards (50%) with the exception of one participant's dataset. Because the respective single subject average had a good signal-to-noise ratio, it was still included in the analysis. Averaging occurred from -100 to 1200 ms with reference to stimulus onset. The 200 ms baseline was set from 100 ms before stimulus onset to 100 ms after stimulus onset, thus including the first 100 ms where both stimuli were identical.

### Data analysis

We compared physically identical stimuli, resulting in two comparisons: iambic deviant–iambic standard (ID-IS) and trochaic deviant - trochaic standard (TD-TS). The difference wave was calculated by subtracting the standard stimulus from the deviant stimulus. This comparison ensures also that any artifact that could possibly arise from the implant in response to the stimulus would be eliminated by the subtraction (see also Lonka et al. (2004) reporting the same approach and Friesen and Picton (2010) reporting successful artifact removal by subtraction for N1 and P2 data). Indeed, no hint of an implant artifact could be detected in the resulting difference waves. The windows of statistical analysis were 50 ms-windows placed around the peaks of the grand average of M2, M4 and M6, that is, the groups that have gained some experience with auditory input by the implant. For the control groups, the grand average determining the windows of analysis included all participants.

The longitudinal data of the implanted children were statistically analyzed by applying a linear mixed effect model with R and the lme4 package (Bates et al., 2014) to account for missing values in the longitudinal datasets. Analysis was performed separately for each stimulus pair and time window with *stimulus* (deviant vs. standard), *duration of implant use* (Mpre, M0, M2, M4, M6), and *electrode* (Fz, Cz, Pz) as fixed effects and *subject* as random effect. Each analysis model was optimized using stepwise backward elimination. The same analysis was performed restricted to the group of congenitally deaf children. For each control group, time window and stimulus pair, a repeated measure ANOVA with *stimulus* and *electrode* as factors was performed. All *post-hoc* multiple comparisons are reported with Tukey-corrected *p*-values, and significance level was set to α = 0.05.

A separate test was run to determine whether the differentiation seen descriptively between the iambic and the trochaic stimuli was also statistically significant. Upon visual inspection, a 100 ms-window was determined for each group, and, analogously to the methods above, a repeated measure ANOVA (for the controls) or a mixed effect model (for the implanted children with *subject* as random effect) was then performed for the factor *condition*.

## Results

**Figure 2:**
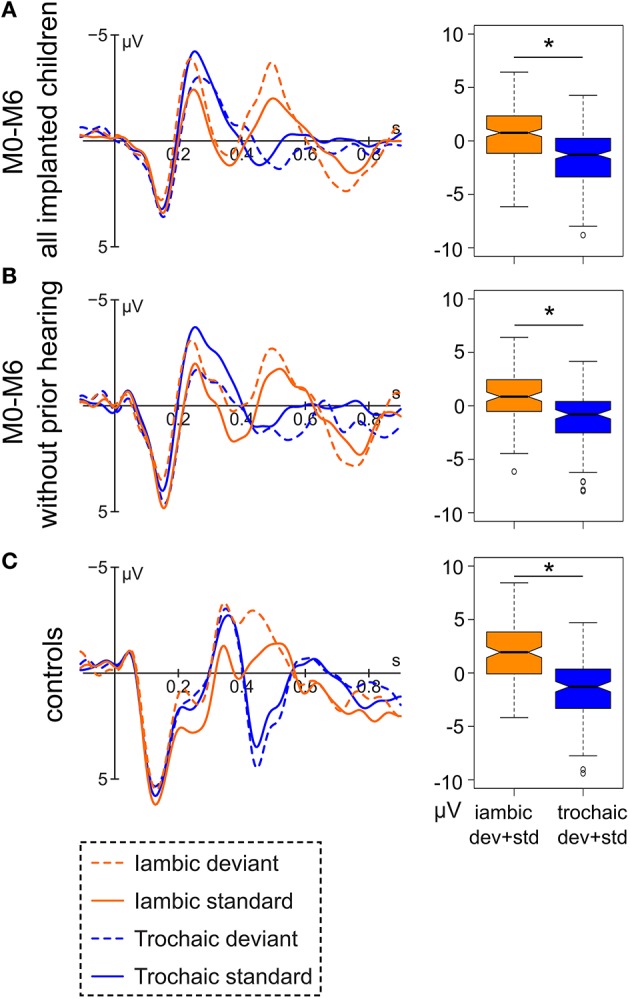
**Left:** Grand average of (A) the data of all hearing-impaired children after implantation (M0-M6; *N* = 47), (B) of the subgroup of congenitally deaf children after implantation (*N* = 19), and (C) all normal hearing controls (*N* = 24) at electrode Fz. **Right:** Respective boxplot representation of the amplitude difference between the iambic and the trochaic stimuli averaged over deviant and standard in the range of 444–544 ms (implanted children) and 404–504 ms (controls). Significant differences between the two stress patterns (*p* < 0.05) are marked by an asterisk.

Both the implanted and the normal hearing group show a clear morphological separation of the trochaic vs. the iambic stimulus around 400–550 ms (Figure 2). The difference was tested for significance in the time window 444–544 ms for the implanted children and 404–504 ms for the control groups. The separate time windows were chosen due to the descriptively clear difference in peak latency between the implanted and the normal hearing group. A shorter ERP latency may be expected for the normal hearing children, as they have a longer hearing experience and thus more mature auditory ERPs. For the group of implanted children, the two stress patterns differ significantly [*F*_(1, 451)_ = 100.99, *p* < 0.001], and the same is observable for the subgroup of congenitally deaf children [*F*_(1, 220)_ = 40.701, *p* < 0.001] and the group of controls [*F*_(1, 286)_ = 115.8, *p* < 0.001].

### Iambic stimulus

**Figure 3:**
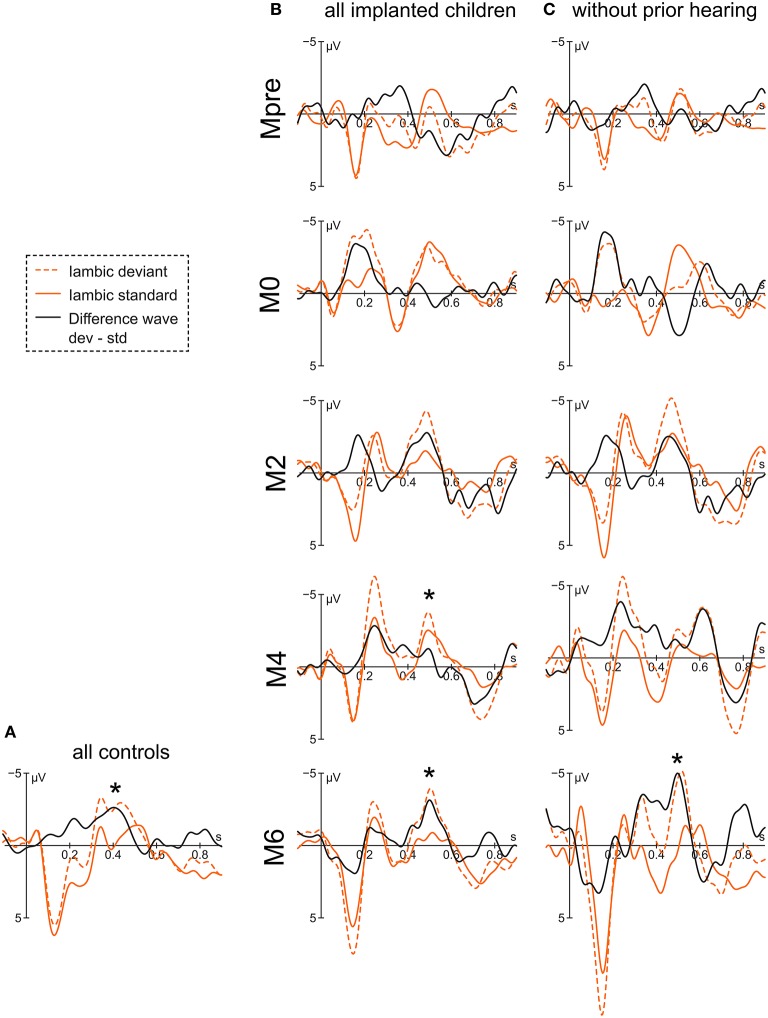
**Grand averages of the iambic stimulus at electrode Fz of (A) the controls, (B) all implanted children, and (C) the congenitally deaf children**. Asterisks mark the significant peak of the difference wave that is assumed to represent the mismatch response.

For the implanted children, the negative peak of the difference wave at 496 ms reveals an interaction of *condition* x *group* [*F*_(4, 253)_ = 4.77, *p* < 0.001] with a significant differentiation between iambic deviant and iambic standard stimulus in the groups Mpre [*t*_(254)_ = −2.29, *p* = 0.02], M4 [*t*_(254)_ = 2.53, *p* = 0.01] and M6 [*t*_(254)_ = 3.71, *p* < 0.001] (Figure 3). In Mpre the effect is due to the deviant stimulus being more positive than the standard stimulus, while in M4 and M6 the difference goes into the opposite direction. The main effect of *electrode* [*F*_(2, 253)_ = 7.15, *p* < 0.001] reveals the negativity to be stronger at Fz compared to Pz [*t*_(254)_ = −3.78, *p* < 0.001]. The subsequent positive peak at 696 ms of the difference wave has a main effect for *condition* [*F*_(1, 259)_ = 16.53, *p* < 0.001] and *group* [*F*_(4, 271)_ = 4.11, *p* = 0.003] with M0 and M4 being significantly more positive than Mpre (M0 vs. Mpre: [*t*_(273)_ = 3.34, *p* = 0.009] and M4 vs. Mpre: [*t*_(273)_ = 2.93, *p* = 0.03], respectively).

When considering only the congenitally deaf children, the difference wave peaks at 488 ms. The significant interaction of *condition* x *group* [*F*_(4, 125)_ = 5.56, *p* < 0.001] reveals a differentiation between the deviant and the standard stimulus only for the group M6 [*t*_(125)_ = 4.24, *p* < 0.001]. A significant main effect of *electrode* [*F*_(2, 125)_ = 3.82, *p* = 0.02] shows a stronger effect at Fz compared to Pz [*t*_(125)_ = −2.64, *p* = 0.03]. The positivity at 704 ms also shows a main effect of *condition* [*F*_(1, 131)_ = 9.83, *p* = 0.002], but is accompanied only by a marginal main effect of *group* [*F*_(4, 135)_ = 2.38, *p* = 0.054], which is due to M2 having more positive values than Mpre [*t*_(136)_ = −2.79, *p* = 0.046].

The two control groups do not differ statistically and are therefore reported together. The control group shows a negative peak in the difference curve at 404 ms with a main effect for *condition* [*F*_(1, 132)_ = 10.67, *p* = 0.001].

### Trochaic stimulus

**Figure 4:**
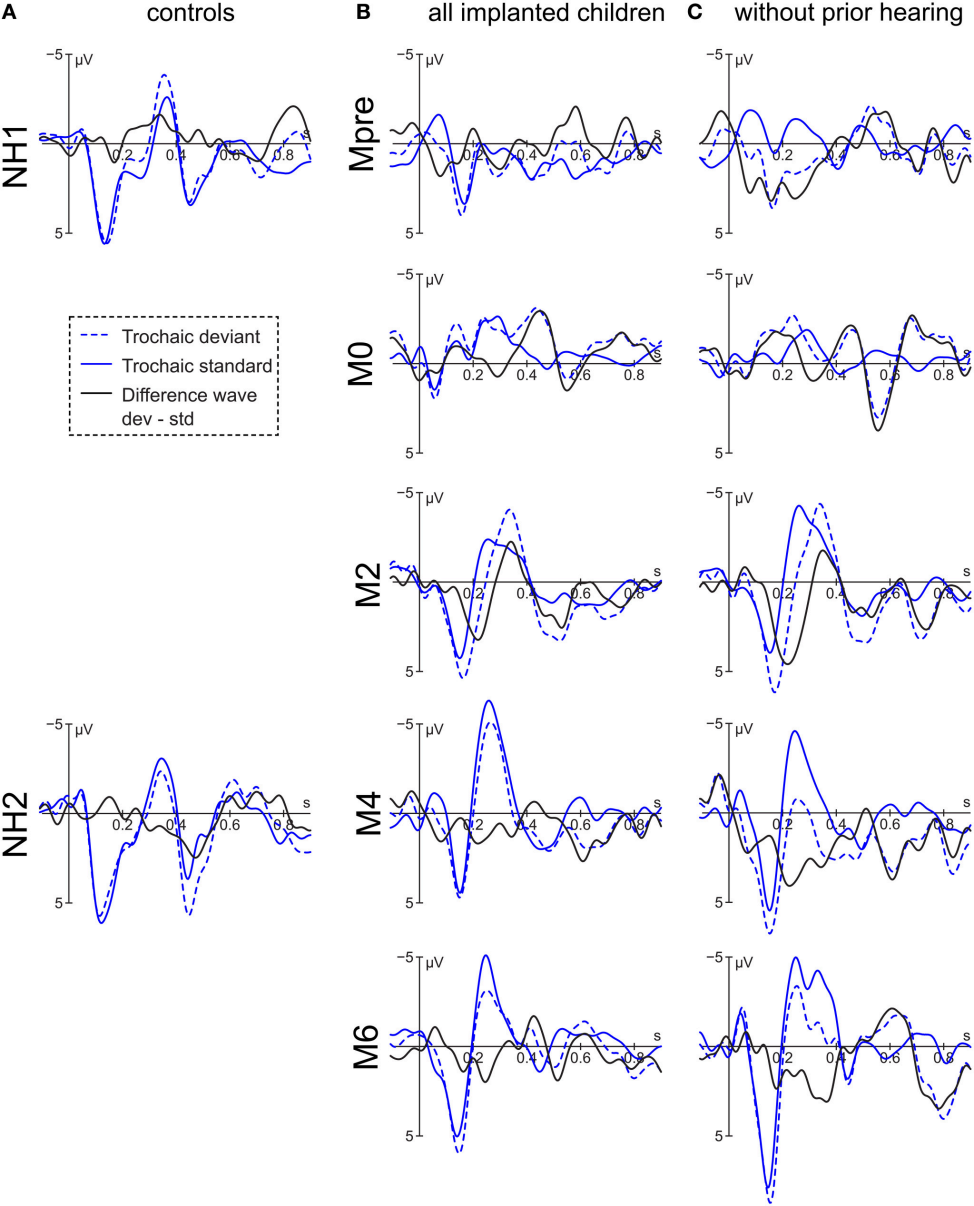
**Grand averages of the trochaic stimulus at electrode Fz of (A) the controls, (B) all implanted children, and (C) the congenitally deaf children**.

For the implanted children, the difference wave of the trochaic stimuli displays a small negativity at 420 ms with significant main effect of *group* [*F*_(4, 270)_ = 4.16, *p* = 0.003] having its source at the larger effect at M6 compared to M4 [*t*_(273)_ = 2.91, *p* = 0.03] and Mpre [*t*_(273)_ = 3.02, *p* = 0.02] (Figure 4). The following positivity at 524 ms shows an interaction of *condition* × *group* [*F*_(4, 256)_ = 2.44, *p* = 0.048) and is driven only by a significant difference at Mpre [*t*_(256)_ = 2.47, *p* = 0.01] where the deviant is, however, more negative than the standard stimulus.

The subgroup of congenitally deaf children displays a negative peak at 504 ms. The negativity reveals a main effect for *group* [*F*_(4, 132)_ = 3.54, *p* = 0.009] with M2 and M4 being more positive than Mpre [*t*_(134)_−3.47, *p* = 0.006] and [*t*_(134)_ = −2.90, *p* = 0.03]. A positive peak is visible at 724 ms, where a significant interaction of *condition* × *group* [*F*_(4, 127)_ = 4.40, *p* = 0.002] is driven by the significant stimulus difference at M4 [*t*_(127)_ = −2.55, *p* = 0.012] and M6 [*t*_(127)_ = −3.63, *p* < 0.001].

The results of the normal hearing peers vary according to group. Whereas NH1 has a small negativity peaking at 480 ms but no significant effect, NH2 has a positive peak at 472 ms with a significant main effect of *condition* [*F*_(1, 66)_ = 4.11, *p* = 0.047] and *electrode* [*F*_(2, 66)_ = 4.86, *p* = 0.01] with Fz being more positive than Pz [*t*_(66)_ = 3.12, *p* = 0.008]. NH1 subsequently presents a negative peak at 836 ms with a significant main effect of *condition* [*F*_(1, 66)_ = 4.94, *p* = 0.03]. NH2 displays a small negative peak at 700 ms with only a marginal effect of *condition* [*F*_(1, 66)_ = 3.79, *p* = 0.056] and a positivity at 896 ms with no significant effect.

## Discussion

Several auditory discriminative abilities are crucial for successful language acquisition. For rhythmic languages like English or German, one of these is the ability to differentiate between categories of stress patterns as the pattern is conveying information about word boundaries. The importance of this ability is highlighted by studies that link a diminished sensitivity to stress pattern deviations with impairments in language acquisition (Friedrich et al., 2009; Goswami et al., 2013). The rationale of the present study was thus to assess, whether the cochlear implant grants children access to the stress pattern information, and, if so, how this discriminative ability evolves over the first 6 months of hearing with the implant.

Most encouragingly, the ERP curves of the implanted children strongly resemble that of normal hearing peers, and this is the case even for the congenitally deaf children. All children show a clear differentiation between the iambic and the trochaic stimuli between 400 and 550 ms. Moreover, between 400 and 500 ms the implanted children respond to a deviant iambic (non-native) stimulus with a significant negative mismatch response (MMR) as do the normal hearing controls. No comparable effect was seen for the trochaic stimulus in either the implanted or the normal hearing children.

The existence of a mismatch response for the foreign iambic but not the native trochaic stimulus parallels the findings of Friederici et al. (2007), where German infants reacted strongly to the non-native iambic stress pattern but not to the native trochaic stress pattern and vice versa for the French infants. It is in contrast, though, to the findings of Segal et al. (2016) who find a stronger reaction toward the native stress pattern, whereas Core et al. (2014) do not differentiate between longer looking times toward the native or the foreign stress pattern.

A further difference to the results obtained by Segal et al. is that while the behavioral study did not find an effect of time with the CI, the ERP data show a development over the first 6 months. The negative MMR for the iambic stimulus is seen descriptively after 2 months of implant use and reaches significance after 4 months. In the subgroup of congenitally deaf children it reaches significance after 6 months of implant use. The latter finding has to be treated with care with regard to the small number of participants in the subgroup, though the small delay of 2 months for the congenitally deaf group is plausible. Considering that those children who had some residual hearing prior to implantation probably have been familiarized to some degree with the native stress pattern prior to implantation, the lag of only 2 months until a significant negative MMR is seen in the congenitally deaf group is remarkably small.

Also remarkable is the fact that even the congenitally deaf children respond with a negative MMR, which is considered to be the more mature mismatch response as opposed to a positive MMR (Kushnerenko et al., 2002; Morr et al., 2002; He et al., 2007, 2009). One could have assumed that, given that their hearing experience is but a few months old, they would display a positive MMR as do the 4- and 5-month-old normal hearing infants in the studies that employed the same stimuli (Friederici et al., 2007). Sharma et al. (2002) have shown that in children that are implanted at a young age (< 6 years) early auditory components like the P1 mature rapidly over the first months of implant use and reach the level of normal hearing peers after 8 months. We observe something similar in our data, though we did not evaluate the P1 component statistically, as it is an early component and we were not sure as to the extent to which it might be altered by an onset artifact of the implant. Descriptively, however, we see a small attenuation of the P1 directly after implantation, followed by a successive increase of amplitude that soon is comparable to those of the normal hearing children. We therefore assume that in our group the long absence of any auditory stimulation causes but a brief delay in catching up with normal hearing peers. That would be in line with our findings on the processing of vowel duration, where congenitally deaf children display ERPs equivalent to their normal hearing peers already after 2 months of implant use (Vavatzanidis et al., 2015).

An important next step would be to increase the group size of implanted children. Recruiting sufficient participants from a clinical group is always a challenge and even more so, when the participants are infants and young children. It would be most valuable, however, to see whether the results can be replicated with a larger group of children. Of particular interest would be a larger group of congenitally deaf children, as they provide a unique insight into (a) the auditory system's performance, when the very first sensory input is delivered with a considerable delay, and (b) how this delay affects further language acquisition.

## Conclusion

This is the first ERP study on stress pattern recognition in implanted children. We demonstrate that the cochlear implant allows the differentiation between native and foreign stress patterns and thus transmits necessary cues for language acquisition. Furthermore we show that even under the condition that there has been no auditory input prior to implantation, young implanted children manage to differentiate between stress patterns within the first 6 months of implant use.

## Author contributions

NV, DM, AF, and AH created the design of the study. AF co-conceptualized a previous form of the paradigm. DM and AF enabled the collection of the clinical and the control data. NV analyzed the data and wrote the manuscript to which all co-authors contributed with critical revisions. AH was also involved to a great extent in data discussion and interpretation. All authors approved the final version and agree to be accountable for this work.

## Funding

Partial funding of this work by the “Marga and Walter Boll Stiftung” (220-02-12).

### Conflict of interest statement

The authors declare that the research was conducted in the absence of any commercial or financial relationships that could be construed as a potential conflict of interest.
